# Myocardial-alternation index (MMI) is correlated with soluble suppression of tumorigenecity-2 (sST2) in patients with ischemic cardiomyopathy

**DOI:** 10.1186/s43044-025-00634-2

**Published:** 2025-04-22

**Authors:** Andra Naufal Pramanda, Fatih Farabi, Hawani Sasmaya Prameswari, Chaerul Achmad, Badai Bhatara Tiksnadi

**Affiliations:** Department of Cardiology and Vascular Medicine, Faculty of Medicine Universitas Padjadjaran, Bandung, Indonesia

**Keywords:** Ischemic cardiomyopathy, Myocardial micro-alternation index, Soluble ST2, Remodeling

## Abstract

**Background:**

Ischemic cardiomyopathy is a condition that represents myocardial dysfunction due to obstructive coronary artery disease. In ischemic cardiomyopathy, both structural and electrical remodeling occur. Myocardial biomarker, soluble ST2 (sST2) is able to predict patient’s mortality and morbidity, and structural remodeling of the heart is responsible for its expression. ECG dispersion mapping (ECG-DM) as evaluated by myocardial micro-alternation index (MMI) may predict alteration of the myocardial electrophysiology with high sensitivity and specificity. The association between structural and electrical remodeling in ischemic cardiomyopathy is not fully understood. This study aims to evaluate the correlation between MMI and sST2 level in patients with ischemic cardiomyopathy.

**Result:**

Total patients who met for the inclusion criteria were 30 patients. Mean age was 57.97 ± 10.04 years; most patients were male (80%). 27 (90%) patients had class II NYHA functional class. The most common risk factors were smoking (20 (66,7%)) and hypertension (17 (56,7%)). Median MMI was 34.0% (IQR: 23.0–42.3%) and median sST2 was 5.6 ng/mL (IQR: 2.0–11.5 ng/mL). This study found that MMI had a significant correlation with sST2, indicating a link between structural and electrical remodeling in ischemic cardiomyopathy (*r* = 0.583, *p* < 0,05).

**Conclusion:**

There was a correlation between MMI and sST2 in patients with ischemic cardiomyopathy.

## Background

The prevalence of patients with ischemic cardiomyopathy is increasing as the number of ischemic heart disease rises. Ischemic cardiomyopathy is the leading cause of heart failure with reduced ejection fraction (HFrEF) (65%) [1]. Patients with ischemic cardiomyopathy had shorter life expectancy and higher admission rate than non-ischemic cardiomyopathy. The most common causes of death in ischemic cardiomyopathy were due to progressive worsening of heart failure and arrhythmias. Both structural and electrical remodeling were responsible for the development of complication in ischemic cardiomyopathy [2].

Several cardiac biomarkers have been reported to be able to assess cardiac structural remodeling in heart failure patients. Among them, American College of Cardiology and American Heart Association Guideline for the Management of Heart Failure stated that soluble ST2 (sST2) and galectin-3, a myocardial fibrosis biomarker, were recommended as class IIb modality for evaluation of patients with acute and chronic heart failure and also for predicting morbidity and mortality of the patients [3]. sST2 belongs to the interleukin-1 receptor family, and its increment may indicate the increase in cardiovascular load and fibrosis. Studies reported that sST2 was associated with higher mortality, worsening of heart failure, reinfarction, and stroke in patients with acute myocardial infarction, acute heart failure, and chronic heart failure [4].

Early detection for electrical remodeling in patients with cardiac disease is essential to decide appropriate treatment. Recently, electrocardiography dispersion mapping (ECG-DM) has been reported as a good modality to assess electrophysiological abnormality of the myocardium with sensitivity of 93% and specificity of 75% [5]. ECG-DM analyzes micro-vibration of the ECG based on the change of amplitude when it approaches a point in which myocardial functional stability is lost. ECG-DM as evaluated by myocardial micro-alternation index (MMI) ranged between 0% and 100%, where 0% represents zero significant alternation which means that each dispersion line is within normal limit. The higher the value of MMI, the greater the deviation of the dispersion line. Alteration of the MMI may reflect alteration of myocardium on metabolic levels that could be caused by ischemia or other pathologic process. Nevertheless, MMI is used as indicator of myocardial electrical alteration rather than diagnostic tool for any cardiovascular disease. [6] This study aims to evaluate the correlation between sST2 and MMI in patients with ischemic cardiomyopathy.

## Methods

### Subjects

This study is a pilot study to determine the association between MMI and sST2 in patients with ischemic cardiomyopathy. The subjects of this study were patients with ischemic cardiomyopathy who were examined at the cardiology polyclinic of Dr. Hasan Sadikin General Hospital, Bandung, between March–May 2022, and were included in the Heart Failure Registry of the Dr. Hasan Sadikin General Hospital, Bandung. Inclusion criteria were patients with ischemic cardiomyopathy of aged ≥ 18 years with left ventricular systolic dysfunction (left ventricular ejection fraction (LVEF) < 40%), New York Heart Association (NYHA) class of II and III, and with 1 or more of the following criteria: [1]

- History of myocardial infarction or revascularization
- Stenosis ≥ 75% in the left main or anterior descending coronary artery
- Stenosis ≥ 75% in 2 or more coronary arteries other than left main and anterior descending.

Patients with primary valvular heart disease, congenital heart disease, acute coronary syndrome, hypertensive heart disease, acute asthma, sepsis, malignancy, systemic lupus erythematosus, and obesity were excluded from the study. Subjects were recruited using a consecutive sampling method. All patients who met the inclusion and exclusion criteria and were admitted to our institution within the specified time period and were enrolled in the Heart Failure Registry were included in this study.

### Study protocol

Data were collected from patients with ischemic cardiomyopathy who came to cardiology polyclinic of the Dr. Hasan Sadikin General Hospital, Bandung, and were included into Heart Failure Registry. Patients who did not have angiography record would be searched on history and evidence of acute coronary syndrome, evidence of non-viable myocardial imaging, and Q pathological on ECG, if the patient had minimal one evidences then he/she would be included. Informed consent was obtained from the patients who met for inclusion and exclusion criteria. The consent letter would be placed in the patient’s medical record and marked as part of the research. After obtaining patient’s consent, we conducted history taking and physical examination. Patients who met the inclusion and exclusion criteria were included to the study and were treated according to the current guidelines for ischemic cardiomyopathy.

For sST2 evaluation, 3 cc of blood was collected from left cubital vein. Samples were then transported to the Clinical Pathology Department in a cooler box at 4°C temperature and centrifuged for 20 min at 1000 g speed at 2–8°C. Samples were stored at − 80–(− 20)°C. When the minimum number of samples was reached, soluble ST2 controls were performed simultaneously. The sST2 assay was performed using the Elabscience® Human sSt2 (Soluble ST2) ELISA Kit.

**Fig. 1:**
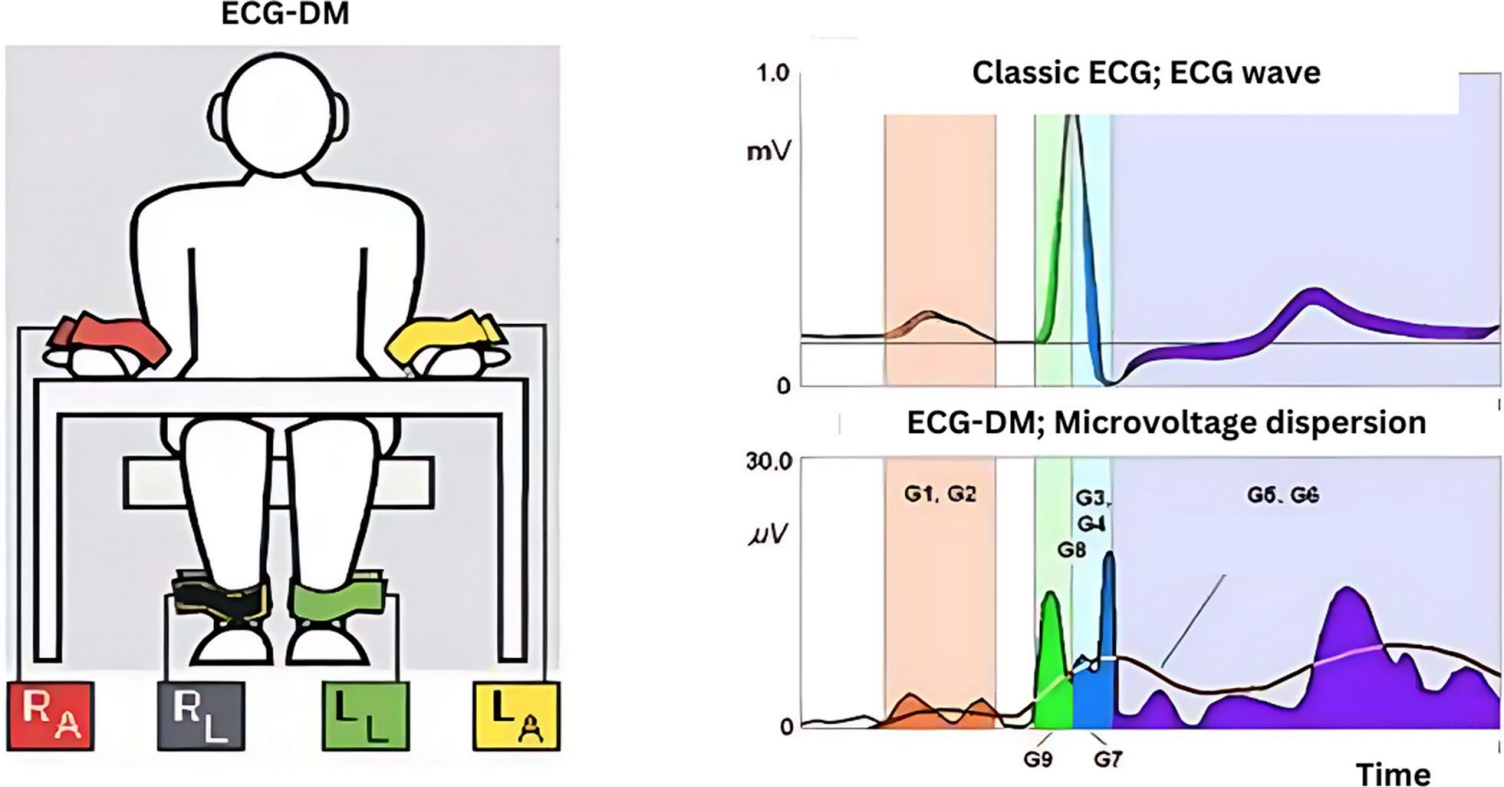
ECG dispersion mapping. G1 and G2: Right and left atrial depolarization (p wave), G9: electrical symmetry, early ventricular asynchronous depolarization (QRS: 0–40 ms), G7: right-left ventricular symmetric depolarization (QRS 30–70 ms), G3 and G4: late right and left ventricular depolarization (late QRS wave: 60–90 ms); G8: right-left ventricular asynchronous depolarization (QRS: 0–90 ms); G5 and G6: right and left ventricular repolarization (ST-T interval). Abbreviation: *RA* right arm, *RL* right leg, *LA* left arm, *LL* left leg (Adapted from CardioDM-06 software for HeartVUE system. User’s manual. 2006)

ECG-DM is an examination to see the micro-vibration amplitude. Dispersion mapping is obtained based on mapping data in changes in the magnitude of electrical micro-voltages from ECG signals; then micro-vibration analysis is performed on changes in amplitude levels when approaching the point where myocardial stability is compromised. Electrophysiological abnormalities in pathological conditions will cause amplitude changes and precede conventional ECG changes. The electrocardiographic characteristics of dispersion mapping are described in 9 analyzed sections, namely G1–G9, which reflect the magnitude and localization of electrophysiological disturbances in the atrial and ventricular myocardium in the depolarization and repolarization periods. In the G1–G9 mapping index, it is assessed in the form of the myocardial micro-alternation index, which has a value range from 0 to 100 percent in assessing the results of deviation or micro-vibratory disturbances. The MMI examination was performed using the HeartVUE® tool. Patients were examined at rest, either sitting or lying down. Patients were asked to take rest for 15–30 min prior to examination. Gel was applied to both wrists and ankles before 4 leads were placed. ECG-DM was recorded for 3 min. Patients were asked to remain still and quiet during the procedure [6] (Fig. 1).

### Statistical analysis

Baseline patient characteristics are presented with categorical data, presented as frequency and percentage, and quantitative data, presented as mean and standard deviation or median and data range (minimum to maximum). Normality was tested using the Shapiro–Wilk test because the number of samples was < 50. Correlation analysis for sST2 and MMI was performed using Spearman correlation test. All statistical tests were performed at 5% significance level.

## Result

### Baseline characteristic

Total patients who had ischemic cardiomyopathy and aged ≥ 18 years who included in Heart Failure Registry were 30 patients. No patients were met for our exclusion criteria; thus, all of them were eligible for final analysis. The mean age was 57.97 ± 10.04 years and 24 (80%) were male. In this study, 27 (90%) patients had NYHA class II, indicating stable cardiac status. All patients in this study had a history of myocardial infarction or acute coronary syndrome. The mean LVEF of the subjects was 31.17 ± 6.24%. Other characteristics of the subjects, including medication use, are shown in the table below (Table 1).

**Table 1:** Baseline characteristics

Characteristics	Total *n* = 30
Age (year), mean ± SD	57.97 ± 10.040
*Sex, n* (%)	
Male	24 (80,0)
Female	6 (20,0)
Body mass index (kg/m^2^), mean ± SD	22.98 ± 3.498
*NYHA functional class, n* (%)	
II	27 (90.0)
III	3 (10.0)
*Risk factor, n* (%)	
Hypertension	17 (56.7)
Diabetes mellitus	7 (23.3)
Dyslipidemia	4 (13.3)
Smoking	20 (66.7)
Family history	2 (6.7)
Menopause	5 (83.3)
*Number of risk factors, n* (%)	
0 risk factor	1 (33.3)
1 risk factor	8 (26.7)
2 risk factors	16 (53.3)
3 risk factors	4 (13.3)
4 risk factors	1 (3.3)
LVEF, mean ± SD	31.17 ± 6.243
PCI, n (%)	20 (95.2)
CABG, n (%)	1 (4.8)
Pathologic Q wave, n (%)	25 (83.3.)
History of revascularization, n (%)	21 (70.0)
*Significant coronary artery lesion, n* (%)	
LM	9 (30.0)
LAD	30 (100.0)
LCx	22 (73.3)
RCA	19 (63.3)
*1 vessel disease*	6 (20.0)
*2 vessel disease*	7 (23.3)
*3 vessel disease*	17 (56.7)
*Pharmacological therapy, n* (%)	
Thrombocyte anti-aggregation	28 (93.3)
ASA	9 (30.0)
CPG	7 (23.3)
ASA + CPG	9 (30.0)
ASA + Ticagrelor	3 (10.0)
Anticoagulant	5 (16.7)
Angiotensin Inhibitor	30 (100.0)
ACE inhibitor	19 (63.3)
ARB	1 (3.3)
ARNI	10 (33.3)
Beta blocker	30 (100.0)
Bisoprolol	30 (100.0)
MRA	29 (96.7)
Statin	30 (100.0)
Furosemide	30 (100.0)
Nitrate	7 (23.3)
MMI (%), median (IQR)	34.0 (23.0–42.3)
sST2 (ng/mL), median (IQR)	5.6 (2.0–11.5)

*SD* standard deviation; *NYHA* New York Heart Association; *LM* left main; *LAD* left anterior descending; *LCx* left circumflex; *RCA* right coronary artery; *RAA* renin angiotensin aldosteron; *ACE* angiotensin-converting enzyme; *ARB* angiotensin receptor blocker; *ARNI* angiotensin receptor neprilysin inhibitor; *MRA* mineralocorticoid receptor antagonist; *CCB* calcium channel blocker; *MMI* myocardial micro-alternation index. *sST2* soluble ST2

## Correlation between MMI and sST2

Median MMI and sST2 of the population are presented in the table below (Table 2).

**Table 2:** Correlation between sST2 and patient’s characteristics

Characteristics	*r*	*P* value
Age (year),	0.137	0.472
Sex	− 0.043	0.822
Body mass index (kg/m^2^),	− 0.081	0.671
NYHA functional class, n	0.035	0.855
*Risk factor*		
Hypertension	− 0.124	0.513
Diabetes mellitus	− 0.032	0.868
Dyslipidemia	− 0.386	**0.035**
Smoking	0.071	0.711
Family history	0.174	0.358
Menopause	0.121	0.819
Number of risk factors, n	− 0.121	0.524
LVEF, (%)	− 0.05	0.792
History of infarction/ACS, n	*	*
History of angiography, n	*	*
Pathologic Q wave, n	0.121	0.524
History of revascularization, n	0.015	0.939
*Vessel disease*	0.0001	1.000
*Pharmacological therapy, n*		
Thrombocyte anti-aggregation	0.028	0.885
Anticoagulant	− 0.005	0.981
Angiotensin Inhibitor	–	–
ACE inhibitor	0.282	0.130
ARB	0.213	0.258
ARNI	− 0.351	0.058
Beta blocker	*	*
MRA	0.241	0.200
Statin	*	*
Furosemide	*	*
CCB	*	*
Nitrate	− 0.178	0.347

*Analysis could not be conducted because all of the subject were within the same group

*NYHA* New York Heart Association; *LM* left main; *LAD* left anterior descending; *LCx* left circumflex; *RCA* right coronary artery; *RAA* renin angiotensin aldosteron; *ACE* angiotensin-converting enzyme; *ARB* angiotensin receptor blocker; *ARNI* angiotensin receptor neprilysin inhibitor; *MRA* mineralocorticoid receptor antagonist; *CCB* calcium channel blocker

**Fig. 2:**
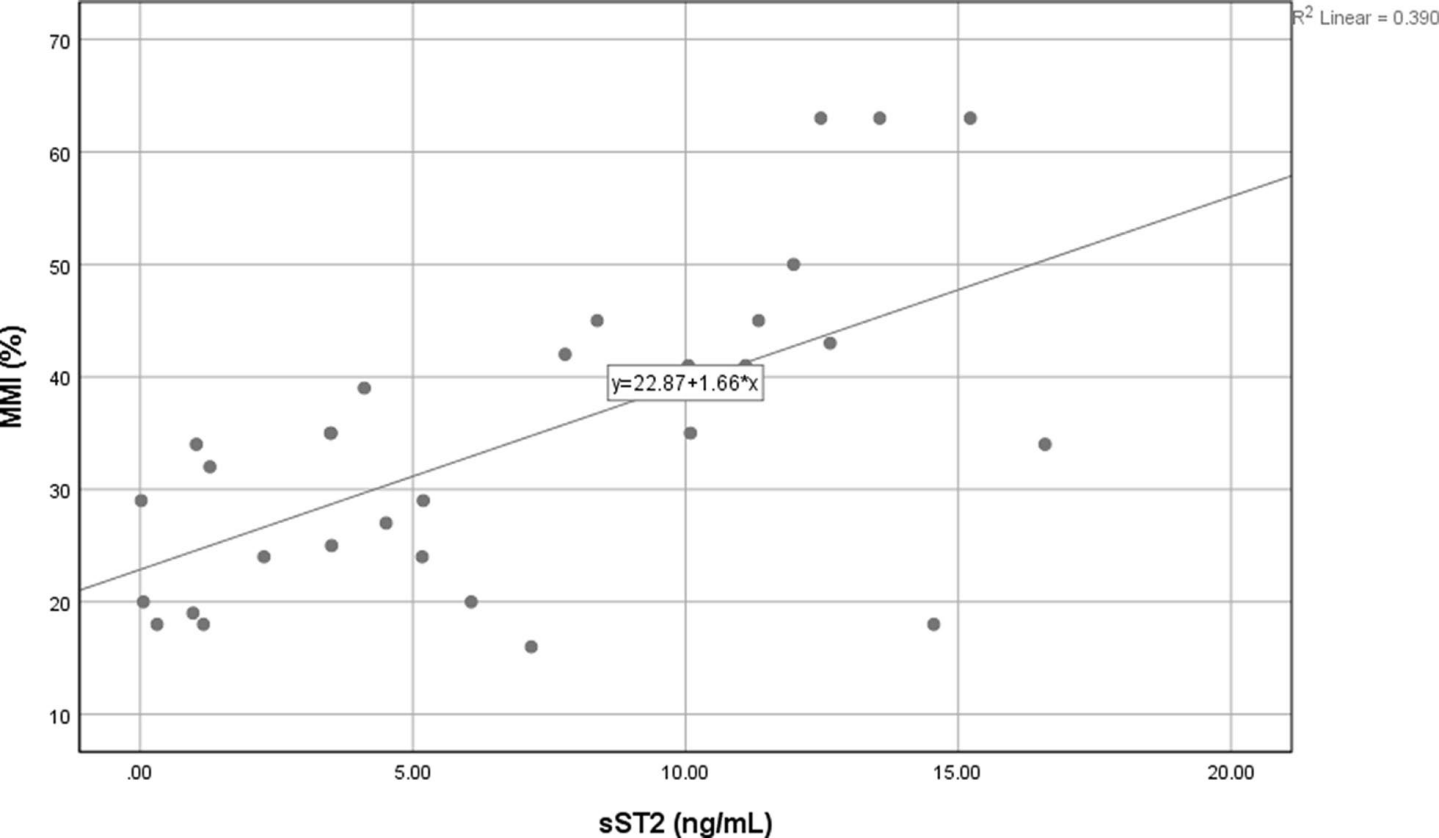
Scatter chart of MMI and sST2. There is a correlation between sST2 and MMI (*r* = 0.583 [95% CI 0.322—0.927], *p* value 0.001). Linear regression showed a positive correlation between these two variables (*p* value < 0.001)

The test showed that the data was not-normally distributed as indicated by *p* < 0.05. Correlation test was then conducted using Spearman test, and we found a significant association with moderate correlation. To evaluate further correlation of MMI and sST2, we performed linear regression test and the result also showed significant correlation. The scatter plot showed an increase in MMI as sST2 increased (Fig. 2).

## Correlation between MMI and sST2 with other characteristics of the subject

We also performed statistical analysis to evaluate if there was any significant correlation between sST2 and MMI with other characteristics of the subjects. The only variable that was found to be significantly associated with sST2 was dyslipidemia with low correlation power (*r* = − 0.386; *p* = 0.035). Other variables that were thought to confound the results of this study such as heart failure medication were not significantly associated with sST2 (Table 2).

We did not find any variable other than sST2 that was significantly associated with MMI. However, the variable with the highest correlation power with MMI was family history of cardiovascular disease (r = 0.325, low correlation power) (Table 3).

**Table 3:** Correlation between MMI and patient’s characteristics

Characteristics	*r*	*P* value
Age (year),	0.022	0.907
Sex, n	0.072	0.704
Body mass index (kg/m^2^),	0.251	0.180
NYHA functional class, n	0.283	0.130
*Risk factor*		
Hypertension	0.066	0.728
Diabetes mellitus	0.100	0.598
Dyslipidemia	− 0.250	0.183
Smoking	0.025	0.898
Family history	0.325	0.080
Menopause	0.131	0.805
Number of risk factors, n	0.192	0.310
LVEF, (%)	− 0.171	0.366
History of infarction/ACS, n	*	*
History of angiography, n	*	*
Pathologic Q wave, n	0.041	0.828
History of revascularization, n	− 0.181	0.338
*Vessel disease*	− 0.023	0.906
*Pharmacological therapy, n*		
Thrombocyte anti-aggregation	− 0.088	0.644
Anticoagulant	0.166	0.382
Angiotensin Inhibitor	–	–
ACE inhibitor	0.205	0.278
ARB	0.183	0.334
ARNI	− 0.213	0.259
Beta blocker	*	*
MRA	0.269	0.151
Statin	*	*
Furosemide	*	*
CCB	*	*
Nitrate	− 0.032	0.867

*Analysis could not be conducted because all of the subject were within the same group

*NYHA* New York Heart Association; *LM* left main; *LAD* left anterior descending; *LCx* left circumflex; *RCA* right coronary artery; *RAA* renin angiotensin aldosteron; *ACE* angiotensin-converting enzyme; *ARB* angiotensin receptor blocker; *ARNI* angiotensin receptor neprilysin inhibitor; *MRA* mineralocorticoid receptor antagonist; *CCB* calcium channel blocker

## Discussion

Ischemic cardiomyopathy refers to reduced pumping ability of the heart due to myocardial changes resulting from ischemic process. In other words, ischemic cardiomyopathy shares similar risk factors with atherosclerosis. They can be divided into modifiable and non-modifiable risk factors. Modifiable risk factors include dyslipidemia, smoking, hypertension, diabetes mellitus, metabolic syndrome, and low physical activity, while non-modifiable risk factors include old age, male sex, and genetics. They all contribute to the pathophysiology of atherosclerosis, which includes endothelial dysfunction, oxidative stress, and lipid accumulation. In addition to atherosclerosis, remodeling processes also contribute to ischemic cardiomyopathy. Risk factors for myocardial remodeling include high hemodynamic stress, neurohormonal activation, and oxidative stress [7]. These reasons may explain the finding in this study that the percentage of risk factors for atherosclerosis and myocardial remodeling was high.

The median sST2 level in this study was 5.6 ng/mL (IQR: 2.0–11.5 ng/mL). A previous study by Broch et al. evaluated sST2 level ​​in ischemic heart failure. The sample population in this study was patients with ischemic heart failure with NYHA class II-IV, LVEF < 40%, and age > 60 years. In the study by Broch et al., out of a total of 1449 patients, the median sST2 level was 17.8 (0.2–400.0) ng/mL [8], while the study by Dimitropoulos evaluated sST2 levels in 143 HFrEF patients with ischemic etiology and found that the mean sST2 was 15.8 ng/mL [9]. This indicates that sST2 levels in this study were much lower than in the ischemic cardiomyopathy population in the other studies. The lower sST2 result compared to other studies may be due to the lower severity of our study population, as 90% of the population classified as NYHA II. The population in this study came from a heart failure clinic where these patients had been receiving guideline-directed medical therapy (GDMT), which was very concerned that although many EFs were low, they could functionally return to NYHA functional class II. In the 2014, the pro-BNP outpatient chronic heart failure therapy (PROTECT) study by Gaggin et al. evaluated the ability of sST2 to identify the risk of left ventricular remodeling in outpatients with low ejection fraction heart failure. This study found that sST2 levels < 35 ng/L had a lower probability of end-diastolic volume index in the first year compared to the group with sST2 levels > 35 ng/mL [10].

Suppression of tumorigenicity 2 is a biomarker of myocardial stress. Weinberg et al. found that transcription of the ST2 gene, especially sST2, was significantly increased in myocytes and cardiac fibroblasts from mice subjected to mechanical stress or myocardial injury in conditions such as myocardial infarction. These results were further confirmed by several in vitro studies reporting similar results [11–13]. Soluble ST2 (sST2) also plays a role in myocardial fibrosis through pathways involving the extracellular matrix. The study by Martinez et al. showed that sST2 was able to stimulate extracellular matrix synthesis by increasing the synthesis of collagen and fibronectin through profibrotic molecules such as TGF-β and connective tissue growth factor (CTGF) [14]. A study conducted by Daniel et al. in 2010 stated that patients with high sST2 levels due to acute myocardial infarction had more significant changes in heart shape than patients with low sST2 levels. Studies suggest that sST2 can be used as a prognostic biomarker to detect heart conditions [15]. Thus, it can be concluded that the heart condition in this study population is better than other studies. This is supported by the finding that the percentage of patients with NYHA class II in this study was higher than in previous studies [90% (this study) versus 68% (Dimitropoulos et al.) and 32.2% (Broch et al.)]. The study by Broch et al. and Dimitropoulos et al. included more patients with NYHA class III and IV than this study [8, 9], but further studies are needed to confirm the exact cause of the low sST2 values ​​in this study. It should be noted that sST2 levels were reported to be unaffected or unrelated to age, body mass index, serum creatinine, and smoking as well as other biomarkers such as CRP, galectin-3, MMP 9, TIMP, and brain natriuretic peptide (BNP). Weinberg et al. in 2013 also stated that sST2 levels did not differ significantly with age, race, and body mass index. Serum sST2 levels were also not affected by the time of collection because the intra-individual biological variation of sST2 was only 11%. Meanwhile, the inter-individual variation of sST2 levels was reported to be 46% with a reference value of 30 [13].

In this study, the median MMI was found to be 34.0% (IQR: 23.0–42.3%). Ivanov et al. performed a screening of DM ECG examination and assessed MMI. In a total of 537,830 people screened in Russia, MMI values were found to be weakly related to gender and age. The study divided the MMI ranges into normal (< 15%), borderline (15–21%), and pathological (> 21%). Similar to this study, we found no correlation between MMI with age and sex in a small number of subjects (*n* = 30). Study by Ivanov et al. found that the prevalence of patients with borderline MMI increased at age 35 years and the prevalence of pathological MMI increased at age 55 years [16]. There are limited studies on MMI measurement in the general population. To best of our knowledge, there have been no studies with English publications evaluating the value of MMI in patients with ischemic cardiomyopathy. However, a study conducted by Bulgakova et al. evaluated the MMI value in patients with acute coronary syndromes. The median MMI value was 24% [17]. Another study evaluated the MMI in acutely ill patients. The study by Kellet et al. found that the mean MMI of acutely ill patients was 23.4%. Kellet et al. also found that MMI was associated with worsening in acutely ill patients [6]. The MMI in these studies was much lower than in this study, because the study population was patients with ischemic cardiomyopathy, who certainly have a higher chronic myocardial remodeling than acutely ill patients and patients with acute coronary syndromes.

The results of correlation analysis using Spearman's rank showed that there was a significant correlation with moderate power between the myocardial micro-alternation index and soluble ST2 (*r* = 0.583, *p* < 0.05). To the best of our knowledge, there has been no study on association between sST2 and MMI in patients with ischemic cardiomyopathy. The association of structural and electrical remodeling in ischemic cardiomyopathy may give clue for better early treatment in such patients. Cardiac remodeling due to fibrosis can be detected by sST2 because of the mechanism described earlier. The process of fibrosis in the heart will affect the flow of electricity in the myocardium, causing arrhythmias. The study by Atabekov et al. found a significant association between sST2 and ventricular arrhythmias in ACS patients with reduced LVEF [18]. Another study by Lashkul et al. found a significant association between sST2 and Left Atrial Volume Index (LAVI) in patients with ischemic cardiomyopathy [19]. It has been known that LAVI was also associated with atrial fibrillation. Another study by Mashlovskyi et al. showed that in NSTEMI patients, higher sST2 levels were associated with the development of ventricular tachycardia and ST segment depression [20]. A study by Skali et al. in 2016 through the Multicenter Automated Defibrillator Implantation Trial (MADIT) evaluated the risk of ventricular arrhythmias in relation to sST2 level in cardiomyopathic patients receiving cardiac resynchronization therapy defibrillator (CRT-D) [21]. sST2 can predict mortality and the incidence of ventricular arrhythmias in patients indicated for CRT-D placement due to ischemic and non-ischemic heart failure. The study also found that serially elevated sST2 levels were associated with a higher risk of ventricular arrhythmias and death or the occurrence of ventricular arrhythmias. In addition, subjects with lower baseline sST2 may benefit more from CRT-D therapy than subjects with higher sST2 [21]. These studies have shown that sST2 is associated with the development of arrhythmias in patients with ischemic heart disease, including ischemic cardiomyopathy. Another study by Broch, et al. in 2016 stated that there was an independent association between sST2 and ventricular arrhythmias in patients with arrhythmogenic right ventricular cardiomyopathy (ARVC). In addition, sST2 also exhibited right-sided heart failure and is independently associated with potentially fatal arrhythmias in this population [8].

Studies on mice by Weinberg et al. and Kakkar et al. have shown that ST2 is associated with myocardial fibrosis and hypertrophy through its interaction with IL-33. Myocardial hypertrophy and fibrosis could reduce cardiac compliance and decrease the diastolic relaxation properties of the heart chambers, leading to disrupted filling of the heart. These conditions indicated that ST2 expression was associated with cell damage and cardiac remodeling which was the pathophysiological basis for cardiomyopathy [13]. In addition to structural remodeling, electrical remodeling also took place in ischemic cardiomyopathy. Hegyi et al. in 2018 conducted a trial by embolization of the porcine anterior descending coronary artery and evaluated the resulting electrophysiological changes at 5 months after infarction. Myocytes in the border zone of the infarct had a shortened action potential duration, whereas myocytes far from the infarct experienced a lengthening of the action potential. These findings suggest that heterogeneous remodeling of the action potential due to ischemia causes a dispersion of action potential duration between the infarct border zone and the remote infarct zone which may be a substrate for arrhythmias. Not only action potentials, but also other heterogeneous electrophysiological remodeling existed between the borderline zone and the far infarct zone such as peak potential, resting membrane potential, maximal upstroke velocity, and maximal rate of repolarization. The finding of their study indicates that ischemic heart failure causes chronic electrical remodeling of the heart. Electrical remodeling contributes to the difference in inflow and outflow of various ions between cardiac cells and ultimately increases the risk of arrhythmia development. However, electrical remodeling in ischemic heart failure is often not detected by ECG, so other modalities that can detect micro-electrical changes in the heart, such as MMI, are needed [22].

The DM ECG method can be referred to as the ECG micro-change recording method. This method is a relatively new non-invasive way of looking at myocardial electrical instability. The amplitude of the micro-changes can be twice as small as the amplitude of the conventional ECG waves.18 One of the assessments in the DM ECG is the MMI. MMI, as previously described, reflects abnormalities in the myocardium at the metabolic rate, which may include ischemia and other causes by detecting micro-alterations in the electrical properties of the heart [6]. In ischemic cardiomyopathy, both structural and electrical remodeling occur. Structural remodeling is characterized by an increase in sST2, whereas electrical remodeling is characterized by changes in MMI. This study found that MMI was significantly correlated with sST2, indicating that there was a correlation between structural and electrical remodeling in ischemic cardiomyopathy. The MMI examination has the advantage of being easy to use and the results obtained are already in the form of numbers, providing a more objective interpretation than other ECG parameters that are operator dependent. The finding of this study may provide evidence for the use of MMI as an effective modality in the risk stratification of patients with ischemic cardiomyopathy. Further studies with larger sample sizes are needed to assess the feasibility of MMI as a risk stratification modality for patients with ischemic cardiomyopathy.

## Limitation

The limitation of this study is the small sample size and classified as a pilot study, which made it difficult to show a strong correlation. However, the result of this study implies that the correlation between MMI and sST2 can be demonstrated even with a small sample size. Therefore, it is suggested that future research be conducted with a larger sample size to validate the results.

## Conclusion

We found a significant association with moderate correlation between MMI and sST2 levels in patients with ischemic cardiomyopathy even in small number of samples. Early detection for electrical remodeling in patients with ischemic cardiomyopathy is essential to decide appropriate treatment. Further studies with a larger number of subjects are needed to assess the feasibility of MMI as a risk stratification modality for patients with ischemic cardiomyopathy. The potential of sST2 as a biomarker needs to be further investigated that describe not only structural, but also cardiac electrical remodeling.

## Data Availability

The authors confirm that the data supporting the findings of this study are available within the article.

## List of abbreviations

ECG-DM: Electrocardiogram dispersion mapping.
MMI: Myocardial micro-alternation index.
sST2: Soluble ST2.
